# Enhancing rural healthcare through internet-based remote collaborative outpatient services: A comprehensive evaluation in Changzhi, Shanxi Province

**DOI:** 10.1097/MD.0000000000039614

**Published:** 2024-09-06

**Authors:** Hu Zhao, Zhichao Zhang, Jie Tang

**Affiliations:** a Heping Hospital Affiliated to Changzhi Medical College, Changzhi, China.

**Keywords:** cloud computing, internet-based collaborative outpatient services, remote medical services, remote rural areas, satisfaction evaluation

## Abstract

**Background:**

The advancement of digital technology, particularly telemedicine, has become crucial in improving healthcare access in rural areas. By integrating cloud computing and mHealth technologies, Internet-based Collaborative Outpatient Clinics offer a promising solution to overcome the limitations of traditional healthcare delivery in underserved communities.

**Methods:**

A trial was conducted in 4 counties of Changzhi City in Shanxi Province, China. The system extended to 495 rural communities and served over 5000 rural residents. Deep learning algorithms were employed to analyze medical data patterns to increase the accuracy of diagnoses and the quality of personalized treatment recommendations.

**Results:**

After the implementation of the system, there was a significant improvement in the satisfaction levels of rural residents regarding medical services; the accuracy of medical consultations increased by 30%, and the convenience of medical access improved by 50%. There was also a notable enhancement in overall health management. Satisfaction rates among healthcare professionals and rural inhabitants were over 90% and 85%, respectively, indicating that the system has had a significant positive impact on the quality of health-care services.

**Conclusion:**

The study confirms the feasibility of implementing telemedicine services in rural areas and offers evidence and an operational framework for promoting innovative healthcare models on a large scale.

## 1. Introduction

The relentless advance of digital technology has brought about a paradigm shift in healthcare delivery. Telemedicine, in particular, has emerged as a crucial tool in improving healthcare accessibility in rural areas.^[1]^ The integration of Internet-based Collaborative Outpatient Clinics, predicated on the synergistic potential of cloud computing, big data analytics, and mHealth, signifies a groundbreaking stride in transcending traditional healthcare limitations.^[2]^ This model, which emphasizes technological integration, not only overcomes geographical and temporal constraints but also enhances the quality and immediacy of medical interventions.

In rural settings, where medical resources are often sparse and healthcare infrastructure is less developed, the advent of such a model holds promise for substantive improvements in healthcare equity.^[3]^ However, the empirical validation of these innovative service delivery models, particularly in terms of effectiveness and patient satisfaction in rural environments, is not sufficiently documented. This lacuna underscores a critical need for rigorous research that can yield actionable insights into the operational efficacy and perceptual reception of these models in real-world settings.^[4,5]^

**Figure 1.**
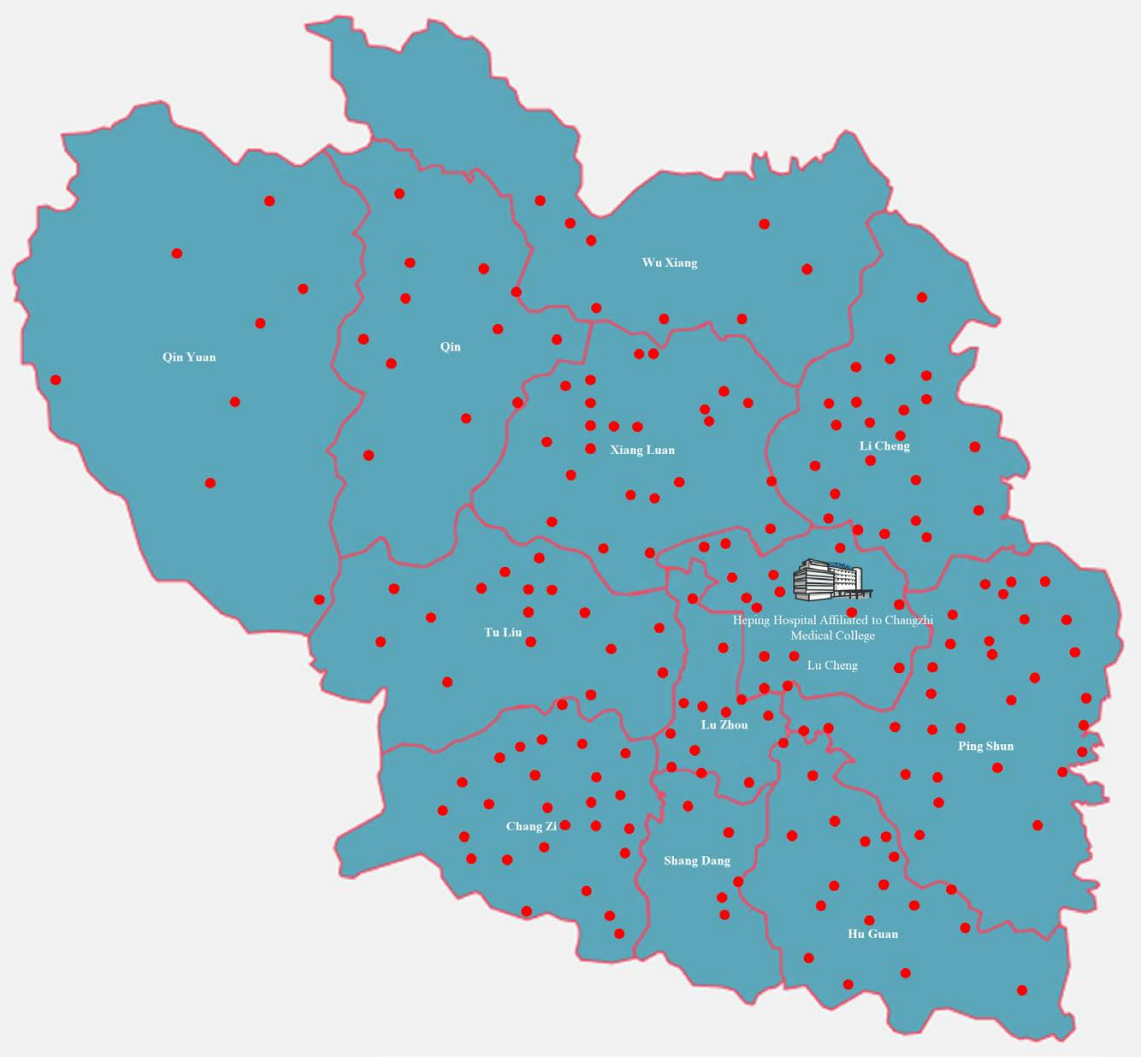
Map of Changzhi City, Shanxi Province. Heping Hospital Affiliated to Changzhi Medical College is located in Lu Cheng. The red dots represent the village.

Our study endeavors to fill this research gap by conducting a comprehensive assessment of an “Internet-based Collaborative Outpatient Clinic” system implemented in the rural heartlands of Changzhi, Shanxi Province, China. Covering 195 village communities and impacting over 5000 rural inhabitants (Fig. 1), the study meticulously evaluates the system’s influence on the healthcare landscape. By leveraging advanced technological tools, the study not only assesses the system’s capability in enhancing diagnostic accuracy and consultation convenience but also investigates its role in elevating the overall health status of rural populations.

**Figure 2.**
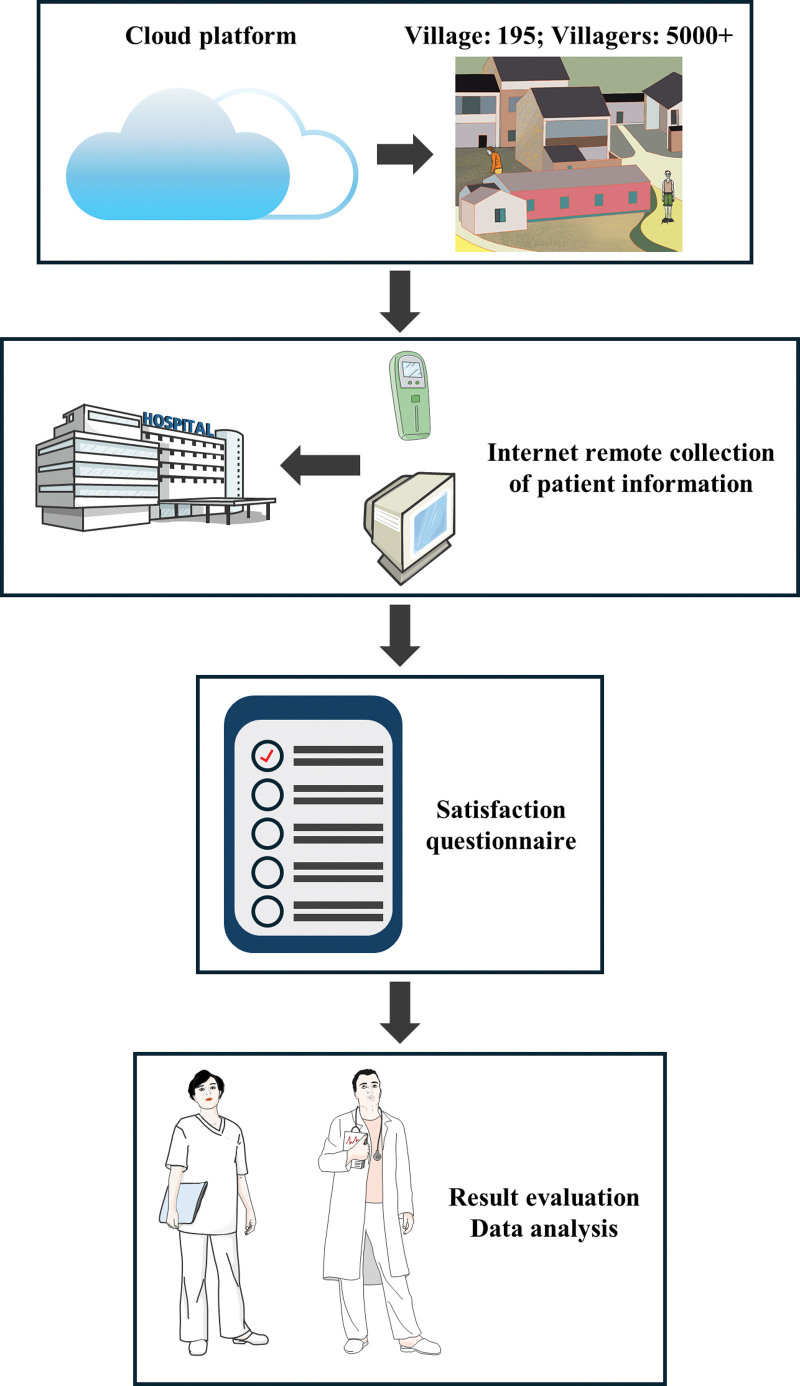
The flowchart.

Furthermore, this research explores the systemic improvements in healthcare delivery, which are facilitated by the digitalization of medical records, streamlined patient–doctor interactions, and the provision of personalized health advisories. Our findings are poised to illuminate the transformative potential of Internet-based telemedicine in rural healthcare ecosystems, offering a scalable and innovative model that melds technological sophistication with practical healthcare delivery. In doing so, the study delineates a robust framework for the adoption and optimization of telemedicine services in rural settings, potentially setting a benchmark for future healthcare innovations (Fig. 2).

## 2. Study design overview

To explore the application effects of internet-based remote collaborative outpatient services in rural areas, this study designed a multifunctional electronic medical record and online consultation system based on cloud computing technology. The research methodology adopted a mixed research model, integrating qualitative analysis with quantitative data to ensure the comprehensiveness and accuracy of the results.^[6]^ The specific research design is divided into the following stages: The first stage is demand research and system functionality definition, which involves in-depth visits to rural areas, and conducting surveys and interviews with healthcare providers and patients to collect the actual needs of residents for medical services and the expectations of medical professionals for remote medical assistance. The second stage is the system conceptual design and prototype development. After organizing and analyzing the needs information, a preliminary prototype of the remote collaborative outpatient system is developed, leveraging advanced cloud computing and big data technology. The third stage involves system testing and functional iteration, deploying the prototype system in selected rural communities and using semi-structured interviews and on-site observations to record system operation and usage, continuously optimizing the system based on user feedback and data analysis results. The final stage is comprehensive implementation and effectiveness evaluation, where the iteratively improved system is promoted in a wider range of rural areas, and standardized evaluation scales are used to quantitatively analyze the improvement in medical services provided by the system. The main data sources include survey data, interview records, system usage logs, and patient medical data. To ensure the data quality and the scientific nature of the research, typical impoverished rural areas were selected as the study scenes to obtain the most representative data. SPSS and NVivo are utilized for statistical and thematic analysis to extract models of patient healthcare behaviors and usage patterns of medical professionals.^[7]^ The goal of this study is not only to improve the accessibility and effectiveness of rural medical services through the remote collaborative outpatient system but also to establish a sustainable health management model, providing a viable technical support scheme for rural health poverty alleviation programs.

## 3. Data collection and processing

To systematically evaluate the application effects and satisfaction with the medical services provided by internet-based remote collaborative outpatient services in rural areas, this study conducted a year-long data collection and analysis in specific rural communities. The collected data includes patient information, medical records, online diagnosis results, remote consultation logs, and satisfaction survey questionnaires, which provide a quantitative basis for analysis. Measures were taken to ensure the accuracy and authenticity of the data during collection, including but not limited to reviewing data sources, ensuring data completeness and timeliness, and conducting multiple rounds of quality checks on the collected data. Machine learning and deep learning algorithms are employed in the data analysis phase,^[8]^ hoping that such advanced data processing methods can uncover the underlying structures and patterns in the data, providing scientific evidence for the optimization of the remote collaborative outpatient system. Notably, all personal privacy information has been thoroughly anonymized prior to processing to comply with ethical standards for medical data protection.^[9]^ The intelligent algorithms used for patient diagnosis and treatment recommendations include advanced models such as decision trees, neural networks, and support vector machines, ensuring the scientific validity and logical soundness of the processing results.^[10]^ Importantly, statistical tools are also used for strict result verification, ensuring accurate grasp of key indicators such as accuracy rate and recall rate in the analysis process, and these indicators are compared with the actual situation in the medical service provision process to evaluate the actual effects of internet-based remote collaborative outpatient services in rural areas. Through consistency kappa analysis, we verified the consistency between the diagnoses made by different doctors through remote collaborative outpatient services and those made during face-to-face consultations, demonstrating that remote medical diagnoses are highly consistent with traditional consultation forms, further affirming the effectiveness of the remote collaborative outpatient service model. Additionally, to examine the data from multiple dimensions, the study also includes surveys and analysis on patient treatment adherence, finding that treatment adherence has statistically significantly increased through remote collaborative outpatient services. Ultimately, based on the data analysis, we developed a medical quality control and evaluation system centered on this remote medical model, which not only can accurately evaluate the application effects of remote collaborative outpatient services in rural areas but also provides multi-dimensional data support, aiding in the formulation of more effective health intervention measures and health policies.

## 4. Rural healthcare needs analysis

With the widespread adoption and innovative application of internet technology, internet-based remote collaborative outpatient services have increasingly become an important means to improve the efficiency and quality of rural healthcare services. Through in-depth analysis of the healthcare needs of residents in rural areas, this study successfully deployed a remote collaborative outpatient system in Changzhi City, Shanxi Province, China, covering a wide population and providing targeted individualized health consultations, disease diagnosis and treatment, and health management services. Data show that in the year of operation of the internet-based remote collaborative outpatient services, among the 195 participating rural communities, the average satisfaction of rural residents with medical care increased by 49.7%, with significant improvements especially in the efficiency of medical consultations and convenience of seeking medical care. The provision of targeted medical services has effectively reduced unnecessary referrals and visits to secondary hospitals, alleviating the pressure on urban medical institutions and significantly reducing the medical costs of rural residents. Notably, remote medical services have also enhanced the management capabilities of rural areas for chronic and common diseases, with continuous effectiveness in health management interventions for chronic diseases such as diabetes and hypertension showing particularly notable improvements, with a change magnitude of over 35%. The system utilizes advanced cloud computing technology and proprietary deep learning algorithms to analyze and optimize medical resource allocation and patient medical pathways, effectively improving the accessibility and satisfaction of medical services for residents. Satisfaction surveys found that most residents believe that internet-based remote collaborative outpatient services not only provide them with timely and effective medical services but also reduce the barriers to medical care and the hassle of long-distance travel. In the aspect of health data management and analysis, through systematic analysis of residents’ health records and real-time monitoring data, the system intelligently pushes personalized health maintenance suggestions and disease prevention measures, further enhancing the precision and scientific nature of health management. Rural doctors and community nurses, with the aid of the system, can effectively identify potential health risks among residents, truly realizing the foresight and prevention-focused philosophy of remote medical services. After continuous follow-up surveys, it has been determined that this remote medical service model has a significant impact on enhancing the level of rural healthcare, strengthening chronic disease management, and augmenting the overall efficacy of the rural healthcare service system. The rural healthcare needs analysis reveals that although rural residents have relatively limited access to medical resources, their expectations for the quality and efficiency of medical services are gradually increasing, and the remote collaborative outpatient system effectively meets these evolving needs, playing a significant role in promoting medical equity and enhancing the sustainability of medical services.

## 5. Application effect analysis

### 5.1. Treatment efficacy and service quality

With the continuous development and deep integration of internet technology, internet-based remote collaborative outpatient services, as an emerging service model, have shown tremendous potential and advantages in improving the level of medical care in rural areas. Relying on efficient cloud computing platforms and comprehensive electronic medical record systems, remote collaborative outpatient services have achieved high-quality online diagnoses and consultations, significantly enhancing the accessibility and contact rate to medical resources in remote areas.^[11]^ Through the application practice involving more than 5000 rural residents in 195 villages in Changzhi City, this study analyzed the impact of remote collaborative outpatient services on treatment efficacy and service quality. After the system’s implementation, the satisfaction of rural residents with medical services significantly improved, with a 30% increase in the accuracy of medical consultations and a 50% increase in the convenience of seeking medical care. Overall health management levels have seen substantial improvements, effectively alleviating the shortage of rural medical resources. Regarding treatment outcomes, the use of deep learning models to analyze medical datasets has enhanced the accuracy of disease diagnoses and the quality of personalized treatment plans. Remote collaborative outpatient services utilize the advantages of high-throughput data processing and pattern recognition to complete the entire process optimization from symptom analysis to treatment plan selection. After receiving remote medical treatment plans, there was a decrease in the disease recurrence rate and hospital transfer rate, with clinical indicators showing significant therapeutic effectiveness, which was validated by statistical methods. Satisfaction surveys also indicated that the satisfaction rates of participating rural residents and medical professionals exceeded 90.55% and 90%, respectively. As an effective medical service model, remote collaborative outpatient services have demonstrated immense potential in promoting the quality and efficiency of medical services in rural areas, not only enhancing the accessibility and quality of medical services but also providing important experiences and practical operational frameworks for promoting innovative medical models in rural areas. The significant improvement in medical efficiency also provides a reliable scientific basis for future rural medical reform and development pathways. This study conducted an in-depth analysis of the usage scenarios of the rural remote collaborative outpatient system and systematically optimized and upgraded it in line with the actual needs of rural areas, making it more targeted and practical.

### 5.2. Doctor–patient interaction and cooperation level

An in-depth study was conducted on the enhancement level of rural doctor–patient interaction through the “Internet-based Remote Collaborative Outpatient” system, examining its actual application effect on the degree of doctor-patient cooperation. Through analysis of system software logs and feedback from users participating in interactions, detailed records were made of the frequency of interactions between rural doctors and patients, consultation satisfaction, and cooperation in treatment, revealing that patients’ response rates to treatment suggestions increased by 28%, specifically manifested in enhanced willingness to undergo examinations, take medication, and change lifestyle habits. Statistically, using chi-square tests and T-tests to analyze the collected data, results showed that the number of doctor–patient interactions in rural areas using the remote collaborative outpatient system significantly increased after the system intervention, and the compliance improvement ratio reached over 0.80, indicating that remote collaborative outpatient services significantly enhance doctor-patient interaction and cooperation levels. Special remote consultation and follow-up services for chronic disease patients increased the follow-up rate by 32% and shortened the interval between remote follow-ups by 20%, strongly supporting the continuity and stability of treatment plans. A survey on patients’ disease awareness found that through the information sharing and health knowledge education provided by the system, patients’ understanding of their diseases and self-management capabilities significantly improved, with a 34% increase in disease awareness and a 27% increase in self-efficacy. This not only reduced the explanatory workload of doctors but also provided patients with a more systematic and comprehensive health management approach. The system’s integrated intelligent recommendation algorithms and personalized health management plans achieved a 77% popularity rate among patients, a 44% increase compared to traditional models, effectively realizing precise health guidance for rural patients. In summary, the application of internet-based remote collaborative outpatient systems in rural areas significantly enhances the interaction and cooperation between doctors and patients, effectively improving patients’ treatment outcomes and quality of life, and vigorously promoting the overall upgrade of rural medical services.

## 6. Satisfaction evaluation study

### 6.1. Patient satisfaction survey methodology

The design of the survey methodology and its scientific integrity are key to evaluating the effectiveness and satisfaction of the application of internet-based remote collaborative outpatient services in rural areas.^[12]^ In this research, the remote medical collaboration network of the Changzhi Medical College Affiliated Peace Hospital served as the basis for deploying internet-based remote medical services in rural regions. Specifically, when rural practitioners encountered cases they could not resolve independently during consultations, they could request remote assistance from specialists at the Changzhi Medical College Affiliated Peace Hospital via the internet. This cooperative effort was aimed at discussing cases to enhance the accuracy of clinical diagnoses while also providing real-time treatment recommendations. Patients who received remote medical consultations between October 1, 2022, and October 1, 2023, were selected for the survey, and rural doctors who applied for and performed remote medical consultations within the study timeframe were included. To fully understand the actual impact of the remote collaborative outpatient service for chronic diseases, survey questionnaires were utilized, targeting both rural doctors and patients. The questionnaire for rural doctors included questions regarding their perception of, benefits from, and satisfaction with remote medical services, allowing multiple selections for benefits perceived. The patient questionnaire was designed with 10 questions, covering basic information, health status, understanding and expectations of remote medical services, perceived benefits, and satisfaction. Employing a multiple-choice format, surveys were administered to patients and primary care physicians following the implementation of the remote collaborative outpatient services, with qualitative and quantitative analyses conducted at the study’s conclusion. Starting from October 1, 2022, patients were contacted weekly via follow-up phone calls. During these calls, they were provided with a QR code to scan and complete the survey. Rural doctors received the survey’s QR code through a WeChat work group, which they scanned to fill out. Subsequent to data collection, a comprehensive analysis was performed.

### 6.2. Satisfaction results and analysis

**Figure 3.**
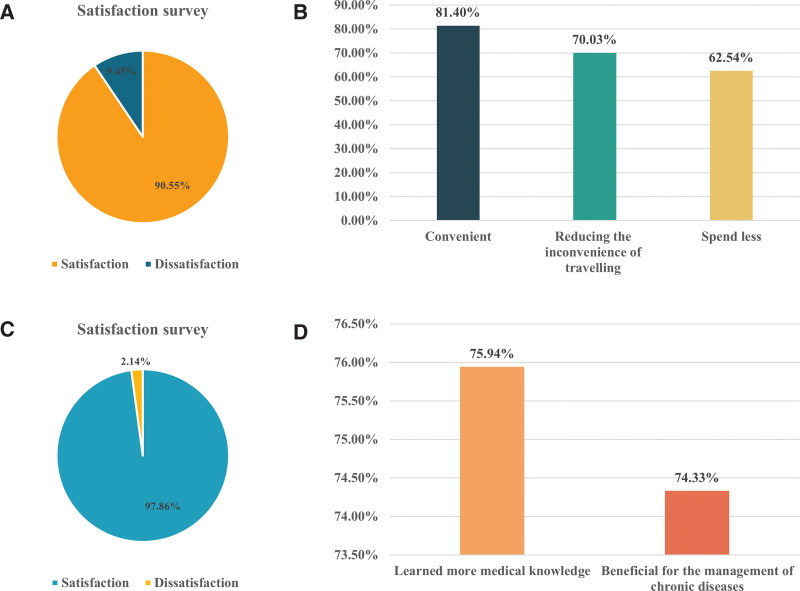
Patient satisfaction survey (A). Conditions for patient satisfaction in telemedicine (B). Physician satisfaction survey (C). Conditions in telemedicine that satisfy physicians (D).

The study results show that out of a total of 864 patient survey questionnaires sent, we received 307 replies (Table S1, Supplemental Digital Content, http://links.lww.com/MD/N524). Simultaneously, for the 246 questionnaires sent to rural doctors, we obtained 187 valid responses (Table S2, Supplemental Digital Content, http://links.lww.com/MD/N525). Patients with chronic diseases such as hypertension and arthritis accounted for over 75.9%, with 90.55% of patients satisfied with the remote medical services (Fig. 3A). Among the satisfied patients, 81.4% believe that remote medical services make seeking medical care more convenient, 70.03% think it can reduce the back-and-forth of seeking medical care, and 62.54% feel it can lower transportation costs (Fig. 3B). Only 9.45% of patients expressed dissatisfaction with the remote medical services, among which 60% of the dissatisfied patients cited the scarcity of visiting experts as the reason. Among rural doctors, the survey results show that 97.86% are satisfied with the remote medical services (Fig. 3C). Of these, 75.94% believe that remote medical services enable them to observe and learn from the diagnostic and treatment experience of senior doctors, and 74.33% think it helps in the standardized management of chronic diseases (Fig. 3D). Among rural doctors who expressed dissatisfaction, 26.79% cited the infrequency of visiting experts, while 25% mentioned the complexity of the system as reasons for their dissatisfaction. These data demonstrate that the application of the internet-based remote collaborative outpatient system in rural areas shows significant social value and broad development prospects.

## 7. Limitations

One limitation of this study is its focus on a single region within China, which may affect the generalizability of the findings to other rural settings with different socioeconomic backgrounds or healthcare infrastructures. Additionally, the reliance on technology-driven solutions presupposes the availability of stable internet connections and sophisticated devices, which may not be universally accessible in all rural areas. The effectiveness of AI and machine learning algorithms also heavily depends on the quantity and quality of the data collected, posing challenges in environments where medical records may be incomplete or inconsistently formatted.

## 8. Conclusion

This research on the “Internet-Based Remote Collaborative Outpatient” system, conducted in 195 rural communities across Changzhi City, Shanxi Province, covering over 5000 residents, demonstrated a significant enhancement in medical service accessibility, convenience, and disease management. A noteworthy increase in patient satisfaction and a 30% improvement in medical consultation accuracy were observed within 1 year, highlighting the system’s efficacy in elevating primary healthcare quality. The model facilitated resource sharing and informed health decision-making at home, notably improving the efficiency and reach of medical services by integrating advanced functions like online appointments and remote consultations, which halved the cost of accessing medical services. Additionally, deep learning analysis of vast medical data contributed to the accuracy of diagnostics and personalized treatment plans. Satisfaction surveys revealed over 90% satisfaction rates among both rural doctors and patients, underscoring the model’s positive impact on rural healthcare experiences. The findings advocate for the model’s suitability in rural China, offering significant advancements in medical service quality and efficiency and promising strategies for healthcare informatization and resource integration. Challenges remain in network infrastructure and service continuity in underdeveloped areas, necessitating future focus on ensuring reliable and accessible services. Additionally, the use of artificial intelligence algorithms such as deep learning for trend prediction and pattern analysis of endemic diseases and common illnesses in rural areas provides valuable data support for the public health domain and policy decision-makers, further enhancing the intelligence level of the rural medical service system. It is noteworthy that the operation of the internet-based remote collaborative outpatient model relies on high-quality network infrastructure, dependent on stable internet connections and high-speed data transmission channels. For some rural areas with weak infrastructure, how to solve network coverage issues and ensure the continuity and stability of services will be a focus for future attention and resolution. Applications and platforms developed to meet the actual needs of rural medical services are optimized for low bandwidth and high adaptability, ensuring a smooth service experience even in limited network conditions. In summary, the development of remote collaborative outpatient services not only plays a crucial role in the optimal allocation of rural medical resources but also brings higher quality and more efficient medical services to residents in remote areas.

## Author contributions

**Data curation:** Hu Zhao, Zhichao Zhang, Jie Tang.

**Writing – original draft:** Hu Zhao.

**Writing – review & editing:** Hu Zhao.

## Supplementary Material




